# Allele-specific binding (ASB) analyzer for annotation of allele-specific binding SNPs

**DOI:** 10.1186/s12859-023-05604-6

**Published:** 2023-12-08

**Authors:** Ying Li, Xiao-Ou Zhang, Yan Liu, Aiping Lu

**Affiliations:** Research Center for Translational Medicine at East Hospital, School of Life Sciences and Technology, Tongji University, Shanghai, 200010 China

**Keywords:** Allele-specific binding, Single nucleotide polymorphism, Motif analysis

## Abstract

**Background:**

Allele-specific binding (ASB) events occur when transcription factors (TFs) bind more favorably to one of the two parental alleles at heterozygous single nucleotide polymorphisms (SNPs). Evidence suggests that ASB events could reveal the impact of sequence variations on TF binding and may have implications for the risk of diseases.

**Results:**

Here we present ASB-analyzer, a software platform that enables the users to quickly and efficiently input raw sequencing data to generate individual reports containing the cytogenetic map of ASB SNPs and their associated phenotypes. This interactive tool thereby combines ASB SNP identification, biological annotation, motif analysis, phenotype associations and report summary in one pipeline. With this pipeline, we identified 3772 ASB SNPs from thirty GM12878 ChIP-seq datasets and demonstrated that the ASB SNPs were more likely to be enriched at important sites in TF-binding domains.

**Conclusions:**

ASB-analyzer is a user-friendly tool that enables the detection, characterization and visualization of ASB SNPs. It is implemented in Python, R and bash shell and packaged in the Conda environment. It is available as an open-source tool on GitHub at https://github.com/Liying1996/ASBanalyzer.

**Supplementary Information:**

The online version contains supplementary material available at 10.1186/s12859-023-05604-6.

## Background

Advances in sequencing technology have led to the identification of widespread variations in the human genome [1]. Some of these variants can lead to cellular and phenotypic changes, thereby contributing to pathogenicity and associated illness. Genome-wide association studies have revealed that most disease-associated variants are located within noncoding regions [2], which comprise approximately 98% of the human genome and contain many transcription factor binding sites (TFBSs) or open chromatin regions [3]. In recent decades, phenotype-related variants have been identified, and a significant number of phenotype-related variants have been implicated to be allele-specific (AS).

AS refers to the phenomenon wherein one allele inherited from either the mother or the father exhibits preferential binding or expression compared to the other allele. Evidence suggests that the AS events are widespread and cell-type specific [4] and can influence many biological processes, such as transcription factor (TF) binding [5–7], chromatin organization [8], RNA binding [9] and gene expression [8, 9]. Allele-specific binding (ASB) is a type of AS event that occurs when transcription factors preferentially bind to one parental allele in a hybrid due to different SNPs inherited from the parental genomes [5]. Likewise, ASB SNPs have been implicated in affecting the expression of downstream effectors by impacting the recruiting efficiency of TFs. As an example, Musunuru et al. [10] reported that the noncoding polymorphism site rs12740374 located at the C/EBP (CCAAT/enhancer binding protein) binding site on 1p13 affected the binding of C/EBP to DNA, thereby altering the expression of the *Sortilin 1* (SORT1) gene and increasing the risk of myocardial infarction. Therefore, ASB analysis provides a unique opportunity to understand the genetic basis of complex traits, including disease susceptibility.

In recent years, there has been a significant interest in associating the identified ASB SNPs with biological processes and clinical manifestations. The typical allele-specific workflow includes preprocessing and alignment of sequencing reads, identification of ASB SNPs, and integration of ASB data with other genomic annotations and functional analysis. Examples of bioinformatics tools developed to identify allele-specific binding SNPs include ABC (allele-specific binding from ChIP-seq) [11], regSNPs-ASB (regulatory SNPs-allele-specific binding tool) [12], BaalChIP (Bayesian analysis of allelic imbalance from ChIP-seq data) [13], GERV (generative evaluation of regulatory variants) [14], ADASTRA (Allelic Dosage-corrected allele-specific human Transcription factor binding sites) [15] and stratAS (stratified allele-specific tool) [16]. Most of these tools aim to minimize issues related to mapping bias in alignment, as the reads carrying the alternative allele may have a lower probability of aligning correctly. After filtering out the mapping bias, allele-specific binding signals can then be identified by comparing the read counts between the parental alleles using either the beta-binomial distribution (e.g., BaalChIP), the negative binomial distribution (e.g., regSNPs-ASB) or other methods, such as learning the effects of specific k-mers on observed binding [14]. However, the aforementioned software programs could not automatically link the ASB SNPs to subsequent biological and clinical analyses, which are of interest to researchers, particularly bioinformaticians. In contrast, ANANASTRA (annotation and enrichment analysis of allele-specific transcription factor binding at SNPs) [17], a web server designed for the annotation and enrichment analysis of allele-specific transcription factor binding at SNPs, could annotate multiple user-submitted SNPs and conduct thorough enrichment analysis, including evaluating ASB SNPs of particular TFs or in specific cell types. Inspired by this software, we next developed an easy-to-use ASB-analyzer pipeline that removes mapping bias with WASP [18] and identifies ASB SNPs using the beta-binomial model. Our multimodular toolkit also automatically connects the identified ASB SNPs to databases such as the Single Nucleotide Polymorphism Database (dbSNP), Genome Browser [19], Variant Viewer [20], the Genotype-Tissue Expression (GTEx) portal [21], and the genome-wide association study (GWAS) catalog [22] to generate an HTML report consisting of a cytogenetic map of ASB SNPs as well as a summary report detailing allele information, read counts, *p* values, crosslinks, features of the cis-regulatory element (cCRE), and motif analysis (Table 1). With this pipeline, we identified 3772 ASB SNPs from thirty downloadable biosamples from The Encyclopedia of DNA Elements (ENCODE) database [23] and demonstrated that the ASB SNPs were more likely to be observed at important sites in TF-binding domains.

**Table 1:** Navigator table interface of ASB SNPs on a chosen chromosome

chrom	Pos	rsID	Counts					cCRE	Motif Enrichment	Genome Broswer link	Variant Viewer link	GTEx	GWAS
			Ref	Alt	Ref	Alt	*p*.val						
chr1	196723542	rs381974	A	G	0	34	4.38E–08	CTCF-only, CTCF-bound	–	Genome Broswer	Variant Viewer	–	–
chr1	111197723	rs599134	C	G	0	22	7.54E–06	dELS, CTCF-bound	YES	Genome Broswer	Variant Viewer	ENSG00000162777.16	Reticulocyte_fraction_of_red_cells
chr1	159821674	rs60205880	G	C	0	21	1.21E–05	CTCF-only, CTCF-bound	YES	Genome Broswer	Variant Viewer	–	–
chr1	153617778	rs9330298	C	A	19	0	3.16E–05	pELS, CTCF-bound	YES	Genome Broswer	Variant Viewer	ENSG00000160678.11	–
chr1	30783058	rs4147103	T	C	2	26	6.78E–05	dELS, CTCF-bound	YES	Genome Broswer	Variant Viewer	ENSG00000162511.7	–
chr1	211649200	rs12741252	G	C	17	0	8.53E–05	dELS, CTCF-bound	YES	Genome Broswer	Variant Viewer	–	–
chr1	89085986	rs11589629	G	C	19	1	0.0003	CTCF-only, CTCF-bound	YES	Genome Broswer	Variant Viewer	ENSG00000213516.9	–
chr1	150067621	rs2027349	A	G	0	9	0.0065	PLS, CTCF-bound	–	Genome Broswer	Variant Viewer	ENSG00000266472.5	–

This is an example of an HTML report for the ASB SNPs located on chromosome 1 (chr1), with the reference SNP identification number (rsID), as indicated. This interactive summary report also lists the position (pos) of an allele, read counts [e.g., reference count (ref count) and alternative count (alt count)], *p* values, cis-regulatory element (cCRE), motif enrichment and analysis, as well as the hyperlinks to multiple databases, including the Genome Browser, Variant Viewer, GTEx portal, and GWAS

## Implementation

**Fig. 1:**
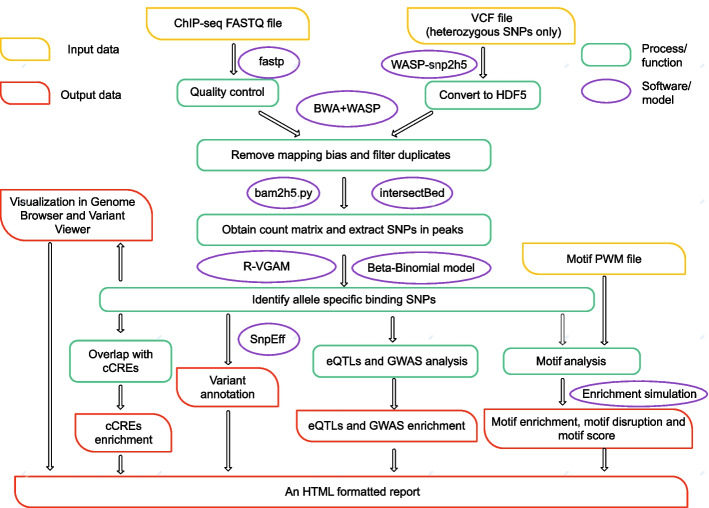
Schematic workflow of the ASB-analyzer. This pipeline is designed to detect, analyze and visualize allele-specific binding (ASB) SNPs. It consists of several steps, initiated by the input data “ChIP-seq FASTQ file”, “VCF file (heterozygous SNPs only)” and “Motif PWM file”, as indicated. The orange rectangles refer to the input data. The purple ovals indicate the software or the model, as indicated, incorporated into the ASB-analyzer multimodular toolkit. The green rectangles denote the specific operational processes or functions included in the package. The red rectangles correspond to the output of those processes or functions

The workflow for our pipeline designed to detect, analyze and visualize allele-specific binding SNPs is illustrated in Fig. 1, and a detailed description of the tools integrated into this pipeline is shown in Additional file 1: Table S1. The proposed ASB-analyzer was initially tested with chromatin immunoprecipitation followed by sequencing (ChIP-seq) datasets of GM12878 downloaded from ENCODE. This ASB-analyzer pipeline utilized input files such as single-end or paired-end ChIP-seq FASTQ files, heterozygous genotype files and the motif position weight matrix (PWM) files to generate an output HTML report containing the ASB SNPs, variant annotation files, and motif analysis results (Fig. 1). Further details are provided in the subsequent sections below.

### ASB SNP identification

The ASB-analyzer workflow is simple with only one manual step (Fig. 1), which requires the users to provide the ChIP-seq FASTQ files in the unmapped read data (.fastq) format, genotype files containing heterozygous SNPs in the Variant Call Format (.VCF) and the corresponding motif PWM file. The input FASTQ files can be either a single-end file or paired-end files (read1 and read2). In the command line, the ChIP-seq data is specified using either the -s or -p flag, where -s denotes single-end data and -p denotes paired-end data. The VCF genotype file could be generated by variant calling software such as Genome Analysis Toolkit (GATK) [24] and SAMtools [25] or by downloading from publicly available databases, such as the 1000 Genome Project [26] or Genome in a Bottle (GIAB) [27] database.

In this pipeline, raw FASTQ files were preprocessed by fastp [28] for quality control with default parameters, while the heterozygous genotypes (.VCF format) were indexed with Tabix and converted to HDF5 format with WASP-snp2h5 script [18]. The FASTQ files were subsequently automatically mapped and remapped using Burrows–Wheeler Alignment (BWA) [29] and then filtered using WASP [18, 30] to remove the mapping bias. Next, Picard [31] was utilized with the default strategy ‘SUM_OF_BASE_QUALITIES’ to filter duplicates. It is worth noting that a read carrying the alternative allele of a variant is considered a mismatch and subsequently discarded due to lower mapping quality (MAPQ). Thus, we specifically incorporated Picard with default parameters, as this collection of command-line tools does not introduce bias due to base quality.

The alleles were then counted using WASP-bam2h5.py [18], and the SNPs were extracted in peaks.

The ASB SNP detection criteria are based on Chen’s method [32], which requires SNP counts to be greater than six, as low counts lack statistical significance. The beta-binomial *p* value was subsequently calculated using the Vector Generalized Linear and Additive Models (VGAM) [33] R package. The expected null distribution assuming no allelic imbalance was then calculated by using the probability density function of the beta-binomial distribution available in the R VGAM. Note that the beta-binomial distribution was defined by the total number of reads at a particular locus, two shape parameters of the beta distribution, and the overdispersion parameter r. Since the null hypothesis assumes no allelic imbalance, the probability of success was fixed at 0.5. Then the expected beta-binomial distributions for r values ranging from 0 to 1 in increments of 0.1 were obtained. The value of r that minimizes the least sum of squared errors (LSSE) between the empirical and expected distributions was selected. Given the read counts for the reference and alternative alleles at a locus, the probability of success (0.5), and the overdispersion parameter (r), the VGAM function pbetabinom could then be used to calculate the *p* value. Finally, an explicit computational simulation was performed to correct for multiple hypothesis testing. The false discovery rate (FDR) was computed by comparing the number of false positives obtained from the simulation to the number of observed empirical positives at a given sliding *p* value threshold. Then, an FDR cutoff of 10% was used for ChIP-seq data, due to the shallower coverage. SNPs that have an FDR below 10% are considered to be ASB SNPs, while those with an FDR above 10% are classified as non-ASB sites.

### ASB SNP function annotation

This section described the steps presented in Fig. 1: “Variant Annotation”, “Overlap with cCREs” and “eQTLs and GWAS analysis”. During “Variant Annotation”, each ASB SNP was annotated with the reference SNP identification number (rsID) and hyperlinked to the corresponding variant information page in dbSNP, Genome Browser and Variant Viewer. The SNP variant annotation and effect prediction tool SnpEff [34] was utilized to obtain information such as genomic regions, base changes, and allele frequency. Genomic region annotated by SnpEff included intron, 3_prime_UTR, 5_prime UTR, downstream_gene, upstream_gene, intergenic_region and other information. Linked expression quantitative trait loci (eQTL) information was annotated using the significant variant-gene associations file downloaded from the GTEx portal. GWAS associations downloaded from the GWAS catalog and incorporated into our pipeline were used to annotate the associated phenotypes.

To further explore the potential genome regulation mechanisms, the ASB SNPs were also mapped to the cCREs. Note that a file containing 1,063,878 human cCREs in GRCh38 downloaded from SCREEN [35] was prebuilt into our pipeline to enable efficient and rapid mapping. Each ASB SNP was labeled with one type of cCRE defined by ENCODE: dELS (distal enhancer-like signature), pELS (proximal enhancer-like signature), PLS (promoter-like signature), DNase-only, CTCF-only, DNase-H3K4me3 and unclassified.

### ASB SNP motif analysis

Motif analysis included three steps as shown in Fig. 1: motif disruption, motif enrichment and motif score. TF-binding motif sequences were scanned by Find Individual Motif Occurrences (FIMO) [36] based on the PWM downloaded from the HOmo sapiens COmprehensive MOdel COllection (HOCOMOCO) database [37]. The FIMO search was incorporated within the scripts of the entire pipeline. Sequences carrying either the reference or alternative allele with ± 20 bp of each SNP were generated in pairs, with each sequence being 41 bp in total and with the SNP at the center. Both the reference and alternate sequences were then scanned for motif prediction. Only the FIMO hits that directly overlapped with the SNP position and had the predicted *p* value less than 1e−4 were considered significant hits.

For each ChIP-seq sample, the statistical significance for the enrichment of the ASB SNPs in the TF-binding motif was estimated. To balance the number of non-ASB SNPs with that of the ASB SNPs and obtain a reliable control group, we randomly selected an equal number of non-ASB SNPs as controls, even though the starting pool of non-ASB SNPs was larger than the number of ASB SNPs. To ensure robustness, this process was iterated 10,000 times to calculate the mean and standard deviation of the non-ASB SNPs located in the TF binding motifs. Subsequently, z scores were calculated and a two-sided *p* value was obtained using the pnorm function in R. Through this analysis, we could determine whether the ASB SNPs significantly disrupted the motif recognition sequences for a particular sample, suggesting a greater impact on regulation.

For each ChIP-seq sample, correlation analyses were performed to examine whether motif disruption by ASB SNPs had a stronger effect than the non-ASB SNPs on TF binding. Within these analyses, two visualization outcomes were generated. The first outcome illustrated the correlation between the allele ratio and the motif score change of the SNPs in motifs. Here, the reference allele ratio is defined as the ratio of reads mapped to the reference allele, while the motif PWM score change is calculated as the PWM score of the reference allele minus that of the alternative allele. The PWM score represents the strength or affinity of a transcription factor's binding to a specific DNA sequence. We then obtained the motif score change and allele ratios for both ASB and non-ASB sites and visualized the data with ggpubr. The Pearson correlation coefficient and corresponding *p* value were calculated using the ggpubr R package. The second outcome showed the position-specific correlation of motif-disrupted SNP frequency with motif conservation, which was measured by the information content (IC). Higher IC indicates that the nucleotide at that position is more conserved and important. Through this analysis, users should be able to determine whether the ASB SNPs in a particular sample are likely to occur at highly conserved sites. The IC was calculated with the R package ggseqlogo, and the Pearson correlation was calculated with R cor.test.

Finally, for batch analysis of multiple samples, we further subdivided the conservation information content and frequency into high and low categories. Specifically, IC and frequency values above the mean values were classified as high, while those below the mean values were classified as low. Additionally, IC and frequency were considered ‘consistent’ when both were correspondingly ‘high’ or ‘low’. Then, the ratio of the consistent positions was calculated.

### Result visualization for each sample

The final step of our analysis involved generating a user-friendly and interactive HTML report summarizing the findings. This report included an interactive and hyperlinked cytogenic map, detailing the distribution of the identified ASB and non-ASB SNPs on each chromosome; the distribution in the different genome regions was annotated by SnpEff. Each ASB SNP, sorted by default using the allele imbalance *p* value, was annotated with the rsID. This HTML report not only included statistical analysis of the motifs, but it also listed the position of the allele, read counts, *p* values, cis-regulatory elements (cCREs), motif enrichment and analysis, and hyperlinks to multiple databases, including the Genome Browser, Variant Viewer, the GTEx portal, and the GWAS database. An example of the HTML formatted output is available in the output summary folder on Github. Overall, our ASB-analyzer’s ability to integrate genotypes with phenotypes is expected to provide researchers with a clearer insight needed to elucidate the potential biological impact of ASB SNPs.

In conclusion, our ASB-analyzer pipeline requires three input files: raw ChIP-seq FASTQ reads, a heterozygous genotype VCF file, and the corresponding motif PWM file. The FASTQ reads and VCF file are used for ASB detection, while the motif file is used for motif analysis. The ASB detection step utilizes the beta-binomial model to output a list of the ASB SNPs. The functional annotation step generates the annotated results by SnpEff and cCREs, along with the distribution bar plots, as well as the hyperlinks to the variant's information page in dbSNP, Genome Browser and Variant Viewer. The motif analysis step provides results on motif enrichment, motif disruption and motif score change. Finally, the results visualization step generates a comprehensive summary of the analysis performed on each ASB SNP. This HTML report includes the basic information of the ASB SNPs, such as the distribution across all the chromosomes, read counts and allele ratios, motif enrichment, eQTLs, and hyperlinks to multiple databases. The HTML report also provides valuable insights into the potential biological significance of these SNPs through GWAS associations (Table 1), the distribution of annotations by SnpEff and the overlap with cCREs, as well as the results of motif analysis.

## Results

### Comparison of mapping bias removal methods

Allelic mapping bias is a known hurdle for the identification of ASB SNPs. However, WASP [18] overcomes this mapping bias by checking the remapping consistency of all reads that overlap the SNP when the allele that is present in the read is changed to match the SNP’s other allele. Correspondingly, hierarchical indexing for spliced alignment of transcripts 2 (Hisat2) [38] overcomes the mapping bias by incorporating a large catalog of known genomic variants and haplotypes into its data structure used for searching and alignment, thereby enabling increased accuracy for the alignment of reads containing the SNPs. To date, no direct comparison of the two methods has been reported.

To search for the best combination of tools to incorporate into our pipeline, we next compared the performance of Hisat2 and BWA, with and without WASP, on various simulated datasets. Specifically, we randomly selected 100 SNPs per chromosome (a total of 2300 SNPs) from NA12878 (also known as GM12878, downloaded from GIAB) to generate the simulated reads. Two types of datasets were constructed, one comprising of single-end (SE) and paired-end (PE) reads with different lengths (e.g., 36 bp, 50 bp, and 100 bp), and the other was more complex, with a fixed read length of 100 bp for both single-end and paired-end reads. In the latter datasets, we also introduced a random mismatch in each constructed read. Additionally, we tested the performance of the different methods when imbalance events occurred by using ratios of reference to alternative alleles with 10% of SNPs between 1.5:1 and 5:1.

**Fig. 2:**
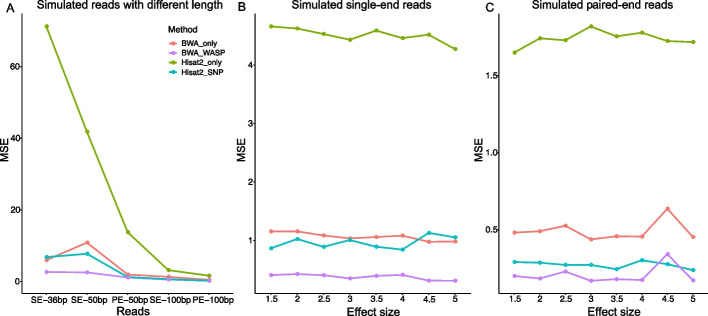
The performance of different methods in removing mapping bias from simulated reads. **A** Performance of Hisat2 or BWA, either alone (e.g., Hisat2-only or BWA-only as indicated) or in combination with WASP (e.g., BWA_WASP) on the simulated reads with different lengths (e.g., 36 bp, 50 bp, and 100 bp, as indicated). Two types of datasets were tested: either single-end (SE) reads with different lengths: SE-36 bp, SE-50 bp, and SE-100 bp; or paired-end (PE) reads with different lengths: PE-50 bp and PE-100 bp. **B** and **C**. Performance of the indicated methods, as detailed in (**A**), on simulated single-end (**B**) and paired-end (**C**) reads with a fixed read length of 100 bp and difference effect sizes. ‘Hisat2_only’ and ‘BWA_only’ referred to methods with basic parameters without removing mapping bias, while ‘Hisat2_SNP’ adopted the parameter of ‘-snp’. Using BWA to map and remap and using WASP to remove bias were indicated with "BWA_WASP". The mean square error (MSE) was used to measure the performance of the different methods. Effect size refers to the ratios of the reference allele and alternative allele with 10% of SNPs between 1.5:1 and 5.0:1

Ideally, the allele ratio of each SNP dataset obtained should be exactly the same as the constructed ratio, but there were some bias in the experimental model due to technical errors. Therefore, the mean square error (MSE) was used to measure the bias of different methods. As shown in Fig. 2, the ‘BWA_WASP’ method, which used BWA to map and remap and WASP to remove bias, outperformed the other methods, yielding the minimum MSE. Therefore, to achieve optimal results, we specifically incorporated the BWA_WASP approach into our pipeline.

### Testing the pipeline using the ENCFF001HIA dataset

**Fig. 3:**
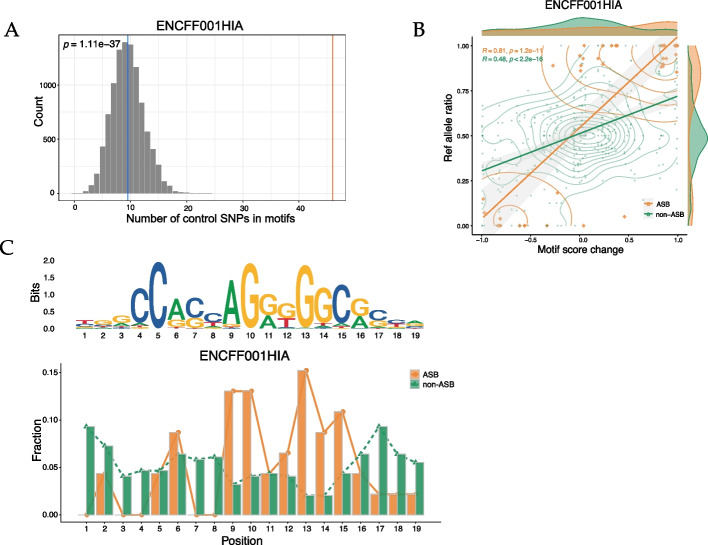
Motif analysis exemplified by ENCFF001HIA. **A** Histogram of the number of ASB SNPs and control non-ASB SNPs in the motifs. The number of ASB SNPs enriched in the motifs is shown as the red line, and the mean value of the control SNPs is shown as the blue line. **B** Scatter plot of the reference allele ratio (Ref allele ratio) and motif score change of the ASB SNPs (ASB, n = 46) and non-ASB SNPs (non-ASB, n = 344). The allele ratio is defined as the ratio of reads mapped to the reference allele, while the motif PWM score change is calculated as the PWM score of the reference allele minus that of the alternative allele. The kernel density plot and the marginal density plot are also shown in the figure. In the kernel density plot, denser contour lines indicate higher data density. The marginal density plot shows the distribution of ASB and non-ASB SNPs in motif score change and reference allele ratio separately. (**C**) Position-specific correlation of motif-disrupted SNP frequency with motif conservation between the ASB and non-ASB SNPs, as measured by the information content (IC), also known as the bit score (Bits)

**Fig. 4:**
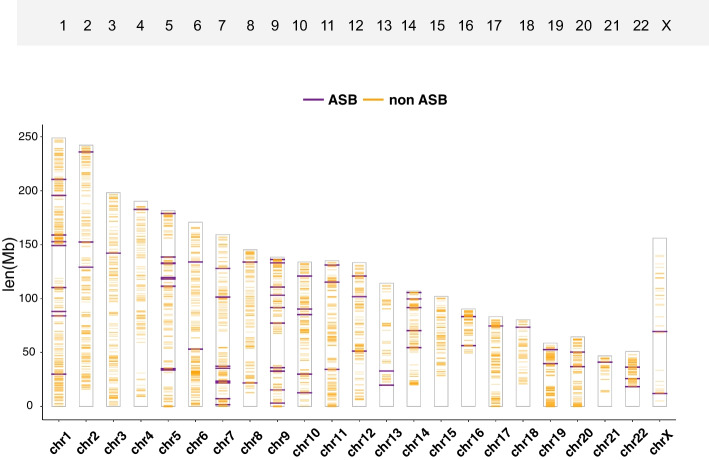
A cytogenetic map of the SNP distribution across chromosomes exemplified by ENCFF001HIA. Distribution of ASB SNPs and non-ASB SNPs in each chromosome. The orange lines represent the non-ASB SNPs, the purple lines represent the ASB SNPs, and “Len (Mb)” corresponds to the genome length in million base-pair

Using the ENCFF001HIA dataset as a test dataset, we first followed the steps detailed in “2.1 ASB SNP identification” to generate a file containing the ASB SNPs, which was subsequently processed following the steps outlined in “2.2 ASB SNP function annotation”. The latter step enabled access to annotation outcomes for the SNPs, enriched cCREs categories, as well as the gene and phenotypic associations among the GTEx eQTLs and GWAS. These phenotype associations were derived from the GWAS catalog database’s data, generated together with the GTEx eQTLs in the “motif/GTEx_GWAS/” folder within the result directory. We then subjected the test dataset to the “2.3 ASB SNP motif analysis” stage, to obtain files and visualizations depicting the results of the motif analysis (Fig. 3). As shown in Fig. 3A, the ASB SNPs for this particular dataset exhibited a marked enrichment within motif recognition sequences when compared to the non-ASB SNPs. Based on comparative analyses, a higher correlation of the reference allele ratio and motif score change were observed in the example data, indicating that the allele-specific events are more likely to occur in the SNPs with larger differences in the motif scores, and that the direction of the imbalance favors the allele with the larger PWM score (Fig. 3B). The position-specific correlation of motif-disrupted SNP frequency with motif conservation, as described in “ASB SNP motif analysis” Section, was further illustrated in Fig. 3C. Our ASB-analyzer thereby supports the premise that, for this specific test dataset, the ASB SNPs are more likely to occur in high information content (IC) positions, indicating a higher likelihood of the ASB SNPs appearing within the conserved regions. For example, in Fig. 3C, ASB SNPs at positions 9, 10, and 13 of the motif exhibit higher information content, suggesting that these positions are more likely to be conserved sites. Here, the approach can be translated to applications on well-established HOCOMOCO [37] TF motifs, as in the case of the Nuclear Receptor Subfamily 2 Group F Member 1 (NR2F1), where the ASB sites follow sequence-specific binding for proteins like the transcription factors and predominantly occurs at key position [39]. To push the analysis one step further, we subsequently subjected the dataset to the steps described in “2.4 Result visualization for each sample”, which generated a comprehensive text file and an HTML-formatted report containing all the discussed information, including the cytogenetic map (Fig. 4) generated by ggplot2 [40] that showed the distribution of ASB SNPs and non-ASB SNPs in each chromosome. All of the information for the ASB SNPs on that chromosome were then displayed (Table 1), just by clicking the corresponding chromosome in the navigation bar. Taken together, our ASB-analyzer combines ASB SNP identification, biological annotation, motif analysis, clinical phenotype associations and report visualization in one pipeline. This composite report should enable the users to speculate on the potential impacts associated with the ASB SNPs of interest. For example, the ASB SNP rs599134, located on chromosome 1, disrupts the motif recognition sequences of CTCF and is associated with glycated hemoglobin levels (Table 1).

### Annotation of ASB SNPs and comparison across samples

A total of 30 datasets of different TF ChIP-seq samples of GM12878 (Additional file 2: Table S2) downloaded from ENCODE were utilized to evaluate the pipeline. In addition to annotating each sample, we also pooled the results of all samples to identify and characterize the more generalized patterns of ASB SNPs.

**Fig. 5:**
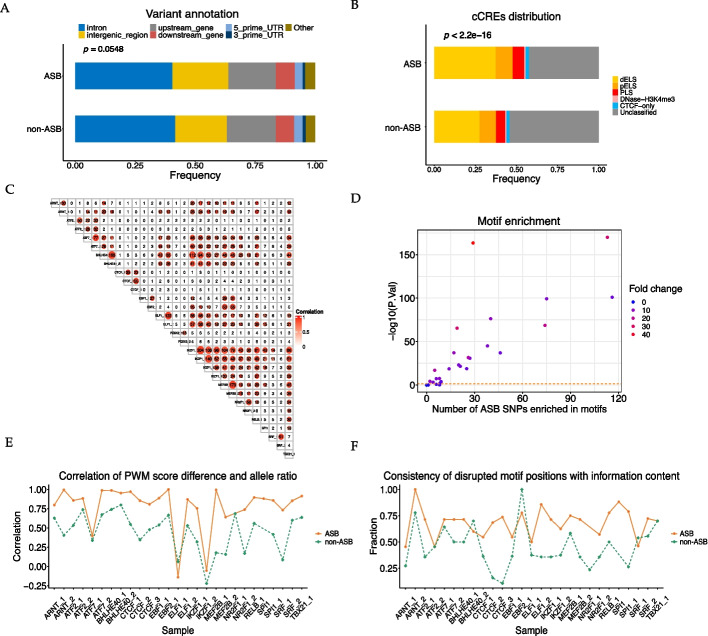
Cumulative results for the thirty ENCODE samples. **A** Distribution of ASB and non-ASB SNPs in different genome regions annotated by SnpEff. **B** Distribution of the ASB and non-ASB SNPs in cis-regulatory elements (cCREs). **C** Pearson correlations of shared SNPs of different samples. **D** Motif enrichment in different samples. The color reveals the fold change in the number of ASB SNPs enriched in motifs and the mean value of controls. **E** Pearson correlations of allele ratio and PWM score difference of the two alleles. **F** Consistency of disrupted motif positions with information content (IC). If the frequency of one SNP’s disrupted location is consistent (either high or low) with its IC, it is ‘consistent’. Higher IC indicates that the nucleotide at that position is more conserved and important. Specifically, information contents and frequencies above the mean values were classified as high, while those below the mean values were classified as low

Genomic annotation performed by SnpEff revealed no significant difference among most of the SNPs located in the intron region, intergenic region and upstream region of a gene (Fig. 5A); the same was observed between the ASB SNPs and the non-ASB SNPs. However, ASB SNPs were significantly enriched in cCREs, especially dELSs (Chi-Squared Test, *p* < 2.2e−16), suggesting that the ASB SNPs might have a stronger effect on genome regulation (Fig. 5B).

The allelic imbalance direction of ASB SNPs across different samples was also compared (Fig. 5C). The allele ratios of shared ASB SNPs between different samples were highly correlated, indicating that the direction of imbalance among the ASB SNPs tended to be consistent across different samples, even though the target TFs were different.

### Impact of ASB SNPs on TF-binding motifs

It has been reported that the ASB events are often associated with changes in TF-binding motifs. Our results confirmed that ASB SNPs were preferentially enriched in the TF motifs compared with non-ASB SNPs. As shown in Fig. 5D and Additional file 2: Table S2, the ASB SNPs were significantly enriched in most TF motifs. Notably, in at least two biological replicates, more than 20 ASB SNPs were enriched in the TF motif sequences for TFs such as CCCTC-binding factor (CTCF), Activating Transcription Factor 2 (ATF2), Basic Helix–Loop–Helix Family Member E40 (BHLHE40), or E74 Like ETS Transcription Factor 1 (ELF1). Correlation analysis of the PWM score change and allele ratio (Fig. 5E) showed that the allele ratio was positively correlated with the difference between the PWM scores of the two alleles, and that the correlation coefficient of the ASB SNPs was higher than that of the non-ASB SNPs. A positive correlation between the PWM score difference and allele ratio was observed in 27 out of 30 samples (Fig. 5E), suggesting that SNPs with a significant difference in PWM scores between the two alleles are more likely to exhibit ASB events. Therefore, the PWM score may be used as a marker for predicting ASB SNPs. Moreover, ASB SNPs were more frequently identified at the conserved sites, as exemplified by the CTCF sample (Fig. 3C) as well as all thirty GM12878 ChIP-seq datasets (Fig. 5F).

## Discussion

Allele-specific binding (ASB) events may play a role in genomic regulation, gene expression and disease susceptibility. Herein, we presented an open-source and standalone pipeline named ASB-analyzer that not only detects ASB SNPs but also annotates and links the identified ASB SNPs to biological and clinical phenotypic alterations. In summary, ASB-analyzer provides a user-friendly platform for efficient identification, characterization, annotation and visualization of ASB SNPs. Its successful application to thirty GM12878 ChIP-seq datasets highlights its effectiveness in uncovering ASB events and their enrichment within transcription factor-binding regions. ASB-analyzer, available on GitHub, serves as a valuable, open-source tool for researchers exploring the genetic nuances of complex traits. The pipeline incorporates scripts in shell, Python, and R codes; it is easily installable on different computers as it is packaged in a Conda environment for managing software dependencies. To the best of our knowledge, our tool is the first pipeline on linux platform that automatically annotates while minimizing mapping bias, and it also provides an interactive and hyperlinked report linking biological phenotypes and disease susceptibility to the ASB SNPs. Like the web-server software ANANASTRA, our tool can provide annotations for dbSNPs and associations with GTEx eQTLs. However, our ASB-analyzer extends beyond these features by offering a more comprehensive range of SNP annotations and analyses. For instance, in addition to dbSNP and GTEx annotations, our platform encompases a more detailed motif enrichment analysis. Our open-source multimodular toolkit also includes information on genomic positional distribution, enrichment in cCREs, and connections to SNPs in the GWAS catalog.

Thirty GM12878 ChIP-seq datasets were used to evaluate the performance of our pipeline. This ASB-analyzer streamlined the whole analytical process, such that only one manual input step is sufficient to generate a detailed HTML summary with informative annotation, plots and hyperlinked resources for each sample. Moreover, the composite statistical analysis of all 30 datasets revealed that the ASB SNPs were enriched in cCREs compared with non-ASB SNPs, especially dELSs (Fig. 5B). Motif analysis further indicated that the ASB SNPs had stronger impacts on TF-binding motifs than the non-ASB SNPs (Fig. 5D–F). In contrast, there was no preference in the distribution of the ASB SNPs in the intronic, intergenic, and upstream regions relative to that of the non-ASB SNPs (Fig. 5A).

One potential limitation of this pipeline is that it currently analyzes ASB SNPs but not allele-specific expression (ASE) SNPs. ASE SNPs are associated with ASB events and have been linked to the occurrence and development of diseases. Therefore, we plan to expand the ASB-analyzer's capabilities to include ASE SNP detection and annotation, developing it into a more flexible and multifunctional toolkit.

## Conclusions

We have developed a novel pipeline that integrates ASB SNP detection, annotation, and visualization in a single package. The mapping bias was minimized by concurrently applying BWA to map and remap and utilizing WASP to filter the bias; the latter step was performed without the phased genotype file. The results from the thirty GM12878 ChIP-seq datasets showed significant enrichment of the ASB SNPs with TF-binding motifs. Additionally, SNPs that are more conserved or exhibit greater differences in PWM scores between the two alleles are more likely to be ASB SNPs.

In conclusion, the ASB-analyzer is a comprehensive and user-friendly pipeline that reports allele counts, allele ratios, motif analysis results, cCRE enrichment, genomic annotation and phenotype associations for each sample. This study provides valuable insights for researchers analyzing the transcriptional regulation and gene expression of ASB SNPs.

## Availability and requirements

Project name: ASB-analyzer.

Project home page: https://github.com/Liying1996/ASBanalyzer

Operating system(s): Linux.

Programming language: Python, R, Bash shell.

Other requirements: Listed on the project home page.

License: MIT license.

Any restrictions to use by non-academics: License needed.

### Supplementary Information

**Additional file 1**. A list of the tools used in this study. This table contains a detailed list of the tools, language/platform, version, as well as the descriptions of the script tools either incorporated into the ASB-analyzer pipeline or used in this work. All listed tools are included in the scripts of the ASB-analyzer pipeline, except for the universalmotif, which was used to calculate the information content of the motifs, as presented in Fig. 5F.

**Additional file 2**. Summary of the motif analysis for the thirty GM12878 ChIP-seq datasets from the ENCODE database. This table lists the sample name (sample) of the thirty GM12878 ChIP-seq datasets as well as their associated TF motifs (TF). It also reports the number of ASB in motif (ASB_inmotif), the total number of ASB SNPs (ASB_total), the number of non-ASB in motif (non-ASB_inmotif), the total number of non-ASB sites (non-ASB_total) for each ChIP-seq dataset as well as the control mean (Control_mean), control standard deviation (Control_sd) and *p* value (P.Val).

## Data Availability

All data generated or analyzed during this study are included in this published article and its supplementary information files. The ASB-analyzer is available as an open-source tool on GitHub at https://github.com/Liying1996/ASBanalyzer.

## Abbreviations

ABC: Allele-specific binding from ChIP-seq
ADASTRA: Allelic dosage-corrected allele-specific human transcription factor binding sites
ANANASTRA: Annotation and enrichment analysis of allele-specific transcription factor binding at SNPs
AR: Allele ratio
AS: Allele-specific
ASB: Allele-specific binding
ASE: Allele-specific expression
ATF2: Activating transcription factor 2
BaalChIP: Bayesian analysis of allelic imbalance from ChIP-seq data
BHLHE40: Basic Helix–Loop–Helix family member E40
Bp: Base pair
BWA: Burrows–Wheeler alignment
C/EBP: CCAAT/enhancer binding protein
cCREs: Candidate cis-regulatory elements
ChIP-seq: Chromatin immunoprecipitation followed by sequencing
chr: Chromosome
CTCF: CCCTC-binding factor
dbSNP: Single nucleotide polymorphism database
dELS: Distal enhancer-like signature
DNase: Deoxyribonuclease
ELF1: E74 like ETS transcription factor 1
ENCODE: The encyclopedia of DNA elements
eQTLs: Expression quantitative trait loci
fastp: FASTQ preprocessor
FASTQ (.fastq): Unmapped read data
FDR: False discovery rate
FIMO: Find individual motif occurrences
GATK: Genome analysis toolkit
GERV: Generative evaluation of regulatory variants
GIAB: Genome in a bottle
GTEx: Genotype-tissue expression
GWAS: Genome-wide association study
H3K4me3: Histone H3 protein with trimethylation at the 4th lysine residue
Hisat2: Hierarchical indexing for spliced alignment of transcripts 2
HOCOMOCO: HOmo sapiens COmprehensive MOdel COllection
IC: Information content
Len (Mb): Genome length in million base-pair
LSSE: Least sum of squared errors
MAPQ: Mapping quality
MSE: Mean squared error
NR2F1: Nuclear receptor subfamily 2 group F member 1
PE: Paired-end
pELS: Proximal enhancer-like signature
PLS: Promoter-like signature
*p* value: Probability value
PWM: Position weight matrix
pos: Position
regSNPs-ASB: Regulatory SNPs-allele-specific binding
rsID: Single nucleotide polymorphism identification number
SAMtools: Sequence alignment/map (SAM) tools
SCREEN: Search candidate cis-regulatory elements by ENCODE
SE: Single-end
SNP: Single nucleotide polymorphism
SnpEff: SNP variant annotation and effect prediction tool
SORT1: Sortilin 1
stratAS: Stratified allele-specific
TF: Transcription factor
TFBSs: Transcription factor binding sites
VCF: Variant call format
VGAM: Vector generalized linear and additive models
